# Uncovering Patient-Reported and Observed Barriers and Facilitators to Sufficient Dietary Intake in Acutely Admitted Older Adults: Insights from a Nutritional Trial

**DOI:** 10.3390/geriatrics11040089

**Published:** 2026-07-17

**Authors:** Olivia Bornæs, Rikke Lundsgaard Nielsen, Ove Andersen, David Peick Sonne, Ingrid Poulsen, Mette Merete Pedersen, Aino Leegaard Andersen

**Affiliations:** 1 Department of Clinical Research, Copenhagen University Hospital Amager and Hvidovre, Kettegaard Allé 30, 2650 Hvidovre, Denmark; rikke.lundsgaard.nielsen@regionh.dk (R.L.N.);; 2 Department of Clinical Medicine, Faculty of Health and Medical Sciences, University of Copenhagen, 2200 Copenhagen, Denmark; 3 Emergency Department, Copenhagen University Hospital Amager and Hvidovre, 2650 Hvidovre, Denmark; 4 Department of Clinical Pharmacology, Bispebjerg and Frederiksberg Hospital, University of Copenhagen, 2400 Copenhagen, Denmark; 5 Department of People and Technology, Roskilde University, 4000 Roskilde, Denmark

**Keywords:** appetite, dietary intake, older adults, nutritional intervention, barriers, facilitators

## Abstract

**Background and Aims**: Insufficient dietary intake is common among hospitalized older (≥65 years) adults with or at risk of malnutrition. Identifying barriers and facilitators is essential for improving nutritional care in this population. This study aimed to identify the most frequently reported and observed barriers and facilitators linked to the achievement of estimated dietary requirements during a nutritional intervention. **Methods**: This descriptive, inductive qualitative content analysis used data from the intervention group (*n* = 65) of the OptiNAM trial (NCT03741283), a randomized controlled trial evaluating a multidisciplinary nutritional intervention among acutely admitted older adults at Copenhagen University Hospital, Hvidovre. A total of 23 of the 65 participants were hospitalized for ≥24 h, allowing dietary intake to be assessed by a dietitian using validated 24 h dietary records, supplemented by dietitian-conducted 24 h recalls when records were unavailable. Individual energy and protein requirements were estimated, and intake was validated and recorded. Meaning units were derived from free-text data from patient interviews or observations during patient interviews that were linked to dietary intake and categorized as barriers or facilitators depending on whether dietary intake was below or above the estimated requirements. **Results**: In acutely admitted older adults with malnutrition or malnutrition risk receiving a nutritional intervention during hospitalization, poor appetite was the most frequently reported barrier to sufficient dietary intake (73.9%), followed by disliking the food (39.1%). In contrast, intrinsic motivation (69.6%), good appetite (34.8%), and support from relatives (30.4%) were the most commonly reported facilitators. **Conclusions**: Appetite, hospital meals, motivation, and support from relatives play central roles in achieving sufficient dietary intake among acutely admitted older adults with or at risk of malnutrition receiving a nutritional intervention.

## 1. Introduction

Insufficient dietary intake is a common challenge among acutely admitted older adults and is associated with adverse outcomes, including prolonged recovery, functional decline, and an increased risk of complications. Consequently, malnutrition and malnutrition risk are highly prevalent in this population, with estimates of up to 60% when using the Mini Nutritional Assessment—Short Form (MNA-SF^®^) as the diagnostic criterion [1]. Despite the implementation of nutritional interventions during acute hospitalization, achieving recommended energy and protein requirements remains challenging. Evidence from intervention studies in older hospitalized adults with malnutrition or malnutrition risk, suggests only partial success in meeting estimated requirements. Our research group has previously conducted a comprehensive nutritional intervention study involving daily individualized dietary counseling by a dietitian, achieving one of the highest levels of dietary coverage reported in the literature, with mean coverage reaching 88.6% (SD 23.1) for energy and 81.8% (SD 23.7) for protein [2]. However, despite this effort, a proportion of patients still failed to meet their individual nutritional requirements, and the underlying mechanisms remain incompletely understood.

Poor appetite is widely recognized as a major barrier to sufficient dietary intake during hospitalization [3], and no effective and satisfactory treatment for poor appetite in malnourished older adults currently exists. Food preferences, food service, and mealtime-related factors have also been reported as important determinants of dietary intake in older adults [4]. These findings have informed the development of nutritional interventions targeting specific aspects of eating behavior and the mealtime environment [5]. However, previous research has primarily examined individual determinants or intervention components, and little is known about the relative importance of multiple patient-reported and observed barriers and facilitators when they coexist in acutely admitted older adults receiving nutritional interventions. Identifying which factors are most commonly associated with insufficient dietary intake despite individualized nutritional support, as well as those that facilitate intake, may help explain why even intensive nutritional interventions often fail to achieve adequate dietary coverage and inform strategies to overcome this challenge.

The aim of this study was therefore to identify patient-reported and observed barriers and facilitators to sufficient dietary intake with regard to the achievement of estimated energy requirements in acutely admitted older adults receiving an individualized nutritional intervention during hospitalization.

## 2. Materials and Methods

### 2.1. Study Design and Participants

This exploratory and descriptive analysis used data from the intervention group (*n* = 65) of the OptiNAM trial, a randomized controlled trial conducted at Copenhagen University Hospital, Hvidovre [6]. For details about the flow of participants in the OptiNAM trial, please refer to Andersen et al. (2024) [2].

Only data from the first five days of the intervention were included. This approach was chosen because nutritional decline may occur rapidly during acute hospitalization, and the first days after admission represent a critical window for establishing sufficient dietary intake. Identifying modifiable barriers during this early phase provides an opportunity to tailor nutritional interventions while patients are still hospitalized, thereby increasing the likelihood of achieving estimated dietary requirements and supporting optimal recovery. The control group was excluded because data relevant to the present analysis were collected exclusively as part of the nutritional intervention delivered to the intervention group.

Participants were adults aged ≥65 years, residing in the Copenhagen area, acutely admitted with a medical illness, and either malnourished or at risk of malnutrition as assessed by the Mini Nutritional Assessment—Short Form (MNA-SF^®^). Only 23 of the 65 participants were hospitalized for ≥24 h, allowing them to be included in this specific analysis. Exclusion criteria included inability to cooperate physically or cognitively, terminal illness, and non-Caucasian ethnicity, the latter due to a substudy examining the applicability of pharmacogenetics for optimizing medication prescribing in older adults, as pharmacogenetic testing may be influenced by ethnicity. Eligible participants were identified through patient records, received written and oral study information, and provided written informed consent. The trial was approved by the Regional Committee on Health Research Ethics (H-18023853) in September 2018 and authorized by the Danish Data Protection Agency (VD-2018-390) in October 2018. The OptiNAM trial was registered at ClinicalTrials.gov (NCT03741283) in November 2018. Further details regarding the study design and methodology have been published elsewhere [6].

### 2.2. Intervention

As this analysis includes only data from the first five days of hospitalization, the description of the dietary intervention provided is confined to this defined study period. An individualized dietary plan was developed by a research dietitian in collaboration with each participant. The dietary plan was based on available hospital meals and nutritional products, individual food preferences, chewing and swallowing ability (assessed using the Eating Assessment Tool-10 questionnaire and, when relevant, by an occupational therapist), and estimated energy and protein requirements. These requirements were determined in accordance with contemporary Nordic and Danish recommendations and were reassessed at each daily visit by the research dietitian, considering body weight, body mass index (BMI), age, physical activity level, and disease-related metabolic stress. The dietary plan could include fixed or à la carte menus, energy- and protein-enriched and/or texture-modified diets, oral nutritional supplements, snacks and protein-enriched beverages between meals, or the protein-enriched liquid supplement Adozan P-Boost^®^ (SanaCare Nutrition ApS, Høsterkøb, Denmark). Dietary intake was recorded daily using a 24 h food diary and reviewed the following day by one of six research dietitians. If the 24 h food diary was incompletely recorded, a 24 h dietary recall was conducted to ensure complete dietary intake data. All dietary intake data and individualized dietary plans were calculated with the web-based VITAKOST software (VITAKOST APS, Kolding, Denmark). If estimated energy and/or protein requirements were not met, the dietary plan was adjusted accordingly. If less than 75% of estimated requirements was achieved for two consecutive days, initiation of enteral or parenteral nutrition was recommended in collaboration with the attending physician.

### 2.3. Variables

#### 2.3.1. Barriers and Facilitators

Factors that hindered the achievement of dietary requirements were defined as barriers, whereas factors that supported their achievement were defined as facilitators. No barriers or facilitators were specified a priori, as the aim was to capture patient-specific factors influencing dietary intake. Barriers and facilitators were identified in daily interviews with the patients in connection with the dietary intake review. During the interview, the dietician recorded both the patient’s reported barriers and facilitators as well as their own observations as free-text data. The two sources of data were thus considered together and were not analyzed as separate data sources. Patient-reported barriers and facilitators were based on patients’ direct responses regarding factors they perceived as influencing their dietary intake. Observed barriers and facilitators were identified by research dietitians based on their clinical assessment of factors likely to influence dietary intake, considering the patient’s condition and circumstances during hospitalization. Observed factors were registered as barriers or facilitators only when they were judged by the research dietitian to have plausible clinical relevance for the patient’s ability to achieve sufficient dietary intake (e.g., tiredness when a participant was observed falling asleep during a meal). Each barrier or facilitator was recorded as a free-text entry only on the first day it was identified. All research dietitians used the same interview approach and registration procedure.

#### 2.3.2. Descriptive Variables

Age and sex were obtained from medical records. BMI was calculated from measured height and weight at baseline. Living condition was self-reported at baseline. Educational level was categorized as ≤14 years of education, skilled training, or >14 years of education. Handgrip strength (HGS), a proxy for physical function according to the European Society for Clinical Nutrition and Metabolism (ESPEN), was measured using a hand dynamometer (Digi-II, Saehan Corporation, Masan, Republic of Korea), with the highest value from three attempts recorded. Need for assistance with grocery shopping, cooking, or eating was assessed using the Functional Recovery Score (FRS). Appetite was assessed using the Simplified Nutritional Appetite Questionnaire (SNAQ), with a cut-off of ≤14 for poor appetite. Nutritional status was assessed using the MNA-SF^®^ and categorized as malnourished (score 0–7) or at risk of malnutrition (score 8–11). Cognitive status was assessed using the Orientation, Memory, and Concentration (OMC) test and categorized as normal cognition (score ≥ 25), mild cognitive impairment (score 18–24), moderate impairment (score 8–17), or severe impairment (score ≤ 7).

### 2.4. Data Analysis

Descriptive variables are presented as means and standard deviations (SD) for continuous variables and as frequencies with percentages for categorical variables. All free-text reports and observations collected during the first five days of hospitalization were analyzed by a project dietitian (OB). A qualitative content analysis with an inductive, data-driven approach was used, with no predefined framework applied. Meaning units were identified, condensed, and coded directly from the free-text data. Codes were subsequently grouped into emergent categories representing barriers and facilitators to sufficient dietary intake. Initial coding and category development were discussed with two members of the research team (MMP and IP), including reflexive discussions regarding the interpretation of codes and emerging categories, to support consistency.

Frequencies of identified barriers and facilitators were calculated descriptively to illustrate how commonly these factors occurred within the study population. These frequencies were used to prioritize factors for discussion and were not interpreted as measures of association or effect.

On day 1, all patient-reported and observed barriers and facilitators were included. On days 2–5, only newly identified barriers and facilitators not previously registered for the individual participant were included, in accordance with the predefined data collection procedure aimed at capturing the emergence of new factors rather than their persistence over time.

Participants achieving ≥75% of their estimated energy requirements on a given day were classified as having sufficient dietary intake, regardless of protein intake. This threshold was chosen as the primary classification criterion because the aim was to identify barriers and facilitators associated with achieving sufficient dietary intake during hospitalization. Energy intake was used as the classification criterion because it provided an objective measure of whether the patient’s overall dietary intake was adequate in relation to their estimated requirements. Protein intake was not included in this classification, as the aim was not to evaluate protein adequacy specifically but rather to explore factors associated with achieving sufficient overall dietary intake. This classification was used to interpret factors that were not inherently considered either barriers or facilitators (e.g., “appetite”), which were categorized according to whether energy intake was below or above this threshold on the corresponding day.

Barriers or facilitators present in ≥30% of participants were prioritized for discussion. This threshold was used as a pragmatic criterion to highlight commonly reported factors and was not intended as an inferential analytical cut-off.

## 3. Results

### 3.1. Participant Characteristics

Among the 65 participants included in the intervention group, 23 participants were hospitalized for at least 24 h following initiation of the nutritional intervention. This allowed for the registration of patient-reported and observed barriers and facilitators, as well as validation of 24 h dietary records or completion of 24 h dietary recalls, and thus their inclusion in the present analysis. Baseline characteristics of the participants are presented in Table 1.

### 3.2. Barriers and Facilitators

A total of 19 barrier categories and 9 facilitator categories were identified. The most frequently reported/observed barrier during the first five days of the intervention was poor appetite (73.9%), followed by disliking food (39.1%). The most frequently reported/observed facilitator was intrinsic motivation (69.6%), followed by good appetite (34.8%) and support from relatives (30.4%). A full overview of all identified barriers and facilitators is presented in Table 2.

## 4. Discussion

### 4.1. Key Findings and Alignment with Previous Studies

Poor appetite emerged as the most important barrier to sufficient dietary intake, consistent with previous findings in similar populations [3], and was closely mirrored by good appetite as the most frequently reported facilitator. The second-most frequently reported barrier, disliking the food, may not necessarily reflect insufficient variety or a lack of energy- and protein-dense options, as the hospital menu offers a wide range of small, energy- and protein-rich meals that can be individually selected. However, visual representation of menu items was absent during hospitalization and may have led to mismatched expectations, potentially reducing meal satisfaction. This would be consistent with previous research emphasizing the importance of food presentation and patient expectations as determinants of dietary intake [7]. It should be noted that altered taste perception may also be influenced by underlying disease, medication, or treatment-related factors [8]. In such cases, even well-prepared and nutritionally adequate meals may be perceived as unappealing, and these challenges may not be easily addressed through food modifications alone.

Intrinsic motivation, good appetite, and support from relatives were identified as key facilitators of sufficient dietary intake. Previous studies have addressed these facilitators in other populations. Behavioral and educational interventions targeting motivation-related constructs such as engagement and self-efficacy in community-dwelling older adults have reduced the prevalence of malnutrition and malnutrition risk [9]. Furthermore, enhancing intrinsic motivation has been associated with improved appetite perception and dietary adherence, particularly when interventions are tailored to individual preferences in older adults [10]. Support from relatives as a facilitator aligns with the existing literature, Vick et al. showed that involving relatives in care planning may strengthen mealtime routines, provide emotional support, and help align hospital nutrition practices with habitual eating patterns in hospitalized older adults [11]. Additionally, a review by Zanetti et al. found that relative involvement can positively influence nutritional status in community-dwelling older adults [12].

### 4.2. Strengths and Limitations

A key strength of the study is the thorough data collection, including the use of individualized nutritional requirements and detailed dietary assessment. Dietary intake was assessed using 24 h dietary records that were validated by a dietitian and complemented by a 24 h recall conducted during a one-to-one intervention session, enhancing the validity and reliability of the data.

This study was exploratory and originally powered based on the primary endpoint of the OptiNAM trial. However, due to the qualitative nature of the method used in this analysis, and because all 23 participants contributed meaningfully and no new categories emerged during the later stages of data collection, the study appears to have reached data saturation. Nevertheless, the relatively small sample size may have limited the ability to capture the full range of barriers and facilitators influencing dietary intake among acutely admitted older adults. Therefore, the findings should be considered hypothesis-generating and interpreted with caution. In addition, the study was conducted at a single hospital, and differences in organizational structures, meal systems, available nutritional support, and patient populations may influence the occurrence and relevance of the identified barriers and facilitators. Thus, the transferability of the findings to other acute care settings and older populations may be limited.

Furthermore, the study population consisted exclusively of Caucasian participants due to the inclusion criteria of the pharmacogenetics substudy from which the sample was derived. Consequently, the findings may not be directly transferable to older adults from other ethnic backgrounds, where cultural factors, food preferences, and meal-related expectations may differ. In addition, patients unable to cooperate cognitively or with terminal illness were excluded to ensure the feasibility of informed consent and data collection. As these patients may represent a particularly vulnerable group with a high risk of insufficient dietary intake, some barriers and facilitators relevant to these populations may not have been captured.

Only participants hospitalized for at least 24 h were included, as a minimum period of hospitalization was required to obtain sufficient dietary intake data and classify whether estimated dietary requirements were achieved. However, this criterion may have excluded patients with shorter hospital stays, who may experience different barriers and facilitators to dietary intake. Additionally, the reduction in the number of participants over the observation period was primarily due to hospital discharge, reflecting the typically short and variable length of stay among acutely admitted older adults. However, patients who remained hospitalized for longer may have differed from those discharged earlier with respect to their clinical condition and nutritional risk. Consequently, findings from the later observation days should be interpreted with caution.

A limitation of the study was that malnutrition was assessed using the MNA-SF^®^, whereas the Global Leadership Initiative on Malnutrition (GLIM) criteria are currently recommended for diagnosing malnutrition. However, as the primary aim of this study was to explore barriers and facilitators for sufficient dietary intake, potential differences in nutritional status classification using GLIM are unlikely to substantially affect the interpretation of the findings. The reported barriers and facilitators are based on participants’ actual dietary intake, which remains relevant regardless of whether individuals would be classified as malnourished according to GLIM. Thus, these insights are still valuable even among patients who may not meet the GLIM criteria for malnutrition.

The definition of sufficient dietary intake was based on achievement of ≥75% of estimated energy requirements and did not include protein adequacy. Consequently, some participants may have been classified as having sufficient dietary intake despite not meeting their protein requirements. This should be considered when interpreting the findings, although the classification was chosen to provide an objective measure of overall dietary intake adequacy in relation to the barriers and facilitators identified.

Although barriers and facilitators were identified using an inductive approach, no formal qualitative framework was applied. Furthermore, the identification and interpretation of observed barriers and facilitators involved clinical judgment by research dietitians, which may introduce researcher-related bias. To reduce this risk, data collection followed a predefined interview procedure, and interpretation of codes and categories was discussed within the research team.

Finally, barriers and facilitators were registered only on the first day they were identified for each participant. Consequently, the present analysis does not capture the persistence of, or changes in individual barriers and facilitators throughout hospitalization. Future studies should investigate how barriers and facilitators develop over time and whether targeted interventions addressing these factors can improve dietary intake.

Despite these limitations, the study provides valuable insights into barriers and facilitators to sufficient dietary intake among acutely admitted older adults receiving a nutritional intervention.

## 5. Conclusions

Poor appetite emerged as the primary barrier to achieving sufficient dietary intake in acutely admitted older adults with or at risk of malnutrition, alongside disliking the food. In contrast, good appetite was the main facilitator, together with intrinsic motivation and support from relatives. These findings suggest that nutritional care should consider both physiological and psychosocial factors influencing dietary intake. However, given the exploratory nature of the study, the relatively small sample size, and the single-center setting, the findings should be interpreted with caution and confirmed in future studies.

## Figures and Tables

**Table 1:** Baseline characteristics.

Characteristic	OptiNAM Intervention Group Hospitalized for ≥24 h (*n* = 23)
Age, years	79.1 (7.2)
Sex, female	14 (60.9%)
Education level	
≤14 years	7 (30.4%)
Skilled	8 (34.8%)
>14 years	8 (34.8%)
BMI, kg/m^2^	24.9 (5.3)
Max hand grip strength, kg	20.1 (5.6)
Living alone	13 (59.1%) *
Needs help with:	
Grocery shopping	11 (47.8%)
Cooking	0 (0%)
Eating	0 (0%)
SNAQ-score ≤ 14	16 (69.6)
MNA-SF^®^	
Malnourished, score 0–7	11 (47.8%)
Risk of malnutrition, score 8–11	12 (52.2%)
Cognitive status (OMC)	
Normal (≥25)	9 (40.9%)
Mild impairment (18–24)	9 (40.9%)
Moderate impairment (8–17)	4 (18.2%)
Severe impairment (≤7)	0 (0%)

BMI = Body Mass Index; OMC = Orientation, Memory, and Concentration; * = 1 missing value; MNA-SF^®^ = Mini Nutritional Assessment—Short Form, SNAQ = Simplified Nutritional Appetite Questionnaire. Values are presented as mean (SD) for continuous variables and as *n* (%) for categorical variables.

**Table 2:** Barriers and facilitators to sufficient dietary intake.

	Day 1	Day 2	Day 3	Day 4	Day 5	Total No.
	*n* = 23	*n* = 21	*n* = 17	*n* = 14	*n* = 8	*n* = 23
**Barriers**						
Poor appetite	14	2	1	0	0	17 (73.9%)
Disliking the food	4	3	1	0	1	9 (39.1%)
Nausea	4	1	0	0	1	6 (26.1%)
Tiredness	2	2	1	0	0	5 (21.7%)
Altered sense of taste	1	0	2	0	0	3 (13.0%)
Pain	1	0	1	0	1	3 (13.0%)
Illness	2	0	0	0	0	2 (8.7%)
Dry mouth	1	0	0	1	0	2 (8.7%)
Bad mood	1	0	0	0	1	2 (8.7%)
Lack of self-awareness of the situation	1	0	1	0	0	2 (8.7%)
Lack of energy	1	0	1	0	0	2 (8.7%)
Decreased quality of life	1	0	0	0	0	1 (4.3%)
Lack of support from relatives	1	0	0	0	0	1 (4.3%)
Risk of refeeding syndrome	1	0	0	0	0	1 (4.3%)
Required fasting	0	1	0	0	0	1 (4.3%)
Received the wrong food	0	1	0	0	0	1 (4.3%)
Lack of intrinsic motivation *	0	1	0	0	0	1 (4.3%)
Vomiting	0	1	0	0	0	1 (4.3%)
Forgetfulness	0	0	1	0	0	1 (4.3%)
**Facilitators**						
Intrinsic motivation *	14	2	0	1	0	16 (69.6%)
Good appetite	4	2	1	0	1	8 (34.8%)
Support from relatives	6	1	0	0	0	7 (30.4%)
Liking the food	5	1	0	0	0	6 (26.1%)
Self-awareness of the situation	3	0	0	0	0	3 (13.0%)
Optimism	2	0	0	0	1	3 (13.0%)
Previous good experience with dietician	2	0	0	0	0	2 (8.7%)
Having energy	0	2	0	0	0	2 (8.7%)
Subsided disease **	0	0	1	1	0	2 (8.7%)

Each observation was recorded only on the first day it was reported or observed. The reduction in *n* from days 1–5 is due to participants being discharged from the hospital. * Intrinsic motivation is defined as motivation arising from the intervention itself or its perceived outcomes, rather than from external factors. ** Subsided disease means that the participant’s disease has improved or become less active.

## Data Availability

The data presented in this study are available on request from the corresponding author. The data are not publicly available due to regulations set out by the Danish Data Protection Agency regarding data anonymization.
